# A Randomised Controlled Trial of Neuronavigated Repetitive Transcranial Magnetic Stimulation (rTMS) in Anorexia Nervosa

**DOI:** 10.1371/journal.pone.0148606

**Published:** 2016-03-23

**Authors:** Jessica McClelland, Maria Kekic, Natali Bozhilova, Steffen Nestler, Tracy Dew, Frederique Van den Eynde, Anthony S. David, Katya Rubia, Iain C. Campbell, Ulrike Schmidt

**Affiliations:** 1 Institute of Psychiatry, Psychology and Neuroscience, King’s College London, London, United Kingdom; 2 Department of Clinical Biochemistry, King’s College Hospital, London, United Kingdom; 3 Neuromodulation Research Clinic, Douglas Mental Health University Institute, Montréal, Québec, Canada; Western Sydney University, AUSTRALIA

## Abstract

**Background:**

Anorexia nervosa (AN) is associated with morbid fear of fatness, extreme food restriction and altered self-regulation. Neuroimaging data implicate fronto-striatal circuitry, including the dorsolateral prefrontal cortex (DLPFC).

**Methods:**

In this double-blind parallel group study, we investigated the effects of one session of sham-controlled high-frequency repetitive transcranial magnetic stimulation (rTMS) to the left DLPFC (l-DLPFC) in 60 individuals with AN. A food exposure task was administered before and after the procedure to elicit AN-related symptoms.

**Outcomes:**

The primary outcome measure was ‘core AN symptoms’, a variable which combined several subjective AN-related experiences. The effects of rTMS on other measures of psychopathology (e.g. mood), temporal discounting (TD; intertemporal choice behaviour) and on salivary cortisol concentrations were also investigated. Safety, tolerability and acceptability were assessed.

**Results:**

Fourty-nine participants completed the study. Whilst there were no interaction effects of rTMS on core AN symptoms, there was a trend for group differences (*p* = 0.056): after controlling for pre-rTMS scores, individuals who received real rTMS had reduced symptoms post-rTMS and at 24-hour follow-up, relative to those who received sham stimulation. Other psychopathology was not altered differentially following real/sham rTMS. In relation to TD, there was an interaction trend (*p* = 0.060): real versus sham rTMS resulted in reduced rates of TD (more reflective choice behaviour). Salivary cortisol concentrations were unchanged by stimulation. rTMS was safe, well–tolerated and was considered an acceptable intervention.

**Conclusions:**

This study provides modest evidence that rTMS to the l-DLPFC transiently reduces core symptoms of AN and encourages prudent decision making. Importantly, individuals with AN considered rTMS to be a viable treatment option. These findings require replication in multiple-session studies to evaluate therapeutic efficacy.

**Trial Registration:**

www.Controlled-Trials.com ISRCTN22851337

## Introduction

Anorexia Nervosa (AN) is a disorder with a high mortality rate and is characterised by a pathological fear of food, eating and gaining weight [1]. In adults with AN, only 10–30% recover with the best available psychotherapies [2–4]. Pharmacological interventions are largely ineffective and have low acceptability [5, 6]. Thus, there is a need to improve treatments [7, 8]. Emerging neuroscience-based technologies that target neural substrates of AN could offer more effective treatment options and may help elucidate disease mechanisms [9–11].

AN is associated with brain changes such as reduced grey matter in fronto-limbic-striatal areas [12, 13]. Functional neuroimaging studies suggest over-representation of limbic drives, e.g. from the insula and amygdala, together with altered prefrontal activity [14]. Alterations in dopamine, 5-hydroxytryptamine (5-HT) and brain-derived neurotrophic factor (BDNF) have also been found i.e. in systems which have been implicated in reward processing, mood and symptom plasticity [15, 16]. Such neuroimaging data have been incorporated into disease models of AN, which suggest altered interactions between ‘bottom-up’ limbic drives (e.g. reward and emotional processing) and ‘top-down’ frontal lobe-mediated cognitive control [17–24].

More specifically, hypoactivity of prefrontal cortex (PFC) regions during response inhibition [25, 26] and set-shifting tasks [27] has been reported in AN. Since set-shifting is assumed to involve the inhibition of a pre-potent rule or behavioural response [28], poor inhibitory control may play a key role in set-shifting difficulties in AN—a well-examined phenomenon that seems to contribute to the maintenance of the disorder. Reduced activity in the PFC may therefore contribute to AN symptoms related to both impaired inhibitory control (i.e. binge eating and purging) and poor cognitive flexibility (e.g. compulsions such as body checking, exercising, and the obsessive pre-occupation with eating, weight and shape).

The dorsolateral prefrontal cortex (DLPFC) has a key role in such self-regulatory control mechanisms and is a common target for neuromodulatory interventions in disorders of fronto-limbic dysregulation. Repetitive transcranial magnetic stimulation (rTMS) applied to the DLPFC is a recognized second-line treatment for depression in the USA [29] and has demonstrated potential in reducing addictive behaviours and craving for nicotine, alcohol and cocaine [30–33]. Similarly, in two sham-controlled trials, our group found that a single session of high-frequency (i.e. excitatory) rTMS over the left DLPFC (l-DLFPC) suppressed food craving in 28 healthy individuals [34] and in 38 patients with bulimia nervosa (BN) [35]. The BN patients also reported fewer binge-eating episodes. Another study did not replicate the effects of rTMS on food craving [36] but was limited by its sample size (*N* = 10) and crossover design. It is important to note that these single-session studies aimed to assess the short-term effects of rTMS and its feasibility as a therapeutic intervention. They are an important first step in informing treatment trials (involving multiple-sessions) that aim to explore long-term clinical efficacy. In a small therapeutic trial (i.e. 15 sessions) of rTMS in 14 individuals with BN, no differences between real and sham rTMS on eating disorder (ED) symptoms or mood were found [37]. Case studies have however, found lasting improvements in symptoms of BN following rTMS treatment [38, 39].

Given its demonstrated efficacy and acceptability in other psychiatric disorders (i.e. BN, addictions and depression), rTMS may be a viable treatment option for AN [40]. Indeed, an uncontrolled case series (N = 10) of a single session of high-frequency rTMS to the l-DLPFC demonstrated short-term reductions in levels of anxiety, feeling full and feeling fat in people with AN [41]. Moreover, case studies/series have found lasting (i.e. up to 12 months) improvements in AN psychopathology and mood following 20 rTMS sessions [42–44].

The physiological and psychological processes underlying the effects of rTMS in AN (and other disorders) are unclear. The DLPFC is implicated in models of emotion regulation [45] and thus rTMS could improve maladaptive emotion regulation strategies in AN (i.e. dietary restraint) [46]. Equally, changes in synaptic plasticity may be involved [47–49], which is in accordance with reported increases in BDNF [47, 50], modulation of extrastriatal dopamine [51] and bilateral DLPFC levels of 5-HT following rTMS [52]. These neural substrates have all been implicated in the aetiopathogenesis of AN [16, 46]. Furthermore, high-frequency rTMS to the DLPFC may remediate hypoactivity within prefrontal brain regions that has been associated with poor inhibitory control [25, 26] and impaired cognitive flexibility [27] in AN.

In psychiatric disorders, including AN, difficulties in inhibitory control have been examined using temporal discounting (TD) tasks, which measure the degree to which a reward is subjectively discounted in relation to its temporal delay. TD tasks provide a measure of choice impulsivity (increased TD i.e. preference for smaller-sooner [SS] rewards) and temporal foresight (reduced TD i.e. a preference for larger-later [LL] rewards) [53]. In a study relevant to AN, healthy participants given a glucose drink demonstrated a preference for LL rewards whilst those given an artificial sweetener drink favoured SS rewards [54]. Given that blood glucose is typically low in AN, these individuals might also demonstrate choice impulsivity. However in AN, TD data are mixed—patients have shown both ‘normal’ [55] and reduced TD behaviour [56, 57]. Interestingly, despite recovered AN patients demonstrating ‘normal’ TD behaviour, task-related functional alterations in the neural substrates of reward and cognitive control (e.g. the DLPFC) are reported to persist [57, 58]. The DLPFC has been implicated in TD [59] and modulation of the PFC by rTMS has been reported to both increase [60] and decrease TD in healthy participants [61, 62]. Given that people with AN may demonstrate aberrant TD behaviour and that rTMS to the PFC has been shown to modulate rates of TD, the present study has explored the effects of rTMS to the l-DLPFC on TD in people with AN.

Lastly, hyperactivity in the hypothalamic-pituitary-adrenal (HPA) axis has been reported in AN [63, 64] and, whilst there were no rTMS-induced changes in our pilot study of AN [41], rTMS has been shown to reduce cortisol concentrations in experimentally-stressed healthy people [65], in depression [66] and in BN [67]. Thus, we have examined the effects of rTMS on cortisol levels in AN.

As discussed previously, single-session rTMS studies a) have demonstrated transient improvements in ED related symptoms [34, 35], b) can be used to elucidate the mechanisms of rTMS and c) are useful in informing future therapeutic trials (re: study design, outcomes, acceptability etc). Therefore, this proof-of-concept study assessed the short-term (i.e. up to 24 hours) effects of a single session of sham-controlled rTMS applied to the l-DLPFC on a) core AN symptomatology, b) TD behaviour and c) stress responses (salivary cortisol). Cardiac safety, tolerability and acceptability of the intervention were also investigated. The primary hypothesis was that real versus sham rTMS would reduce core AN symptoms. Secondary hypotheses were that, compared to sham, real rTMS would improve other psychopathology (anxiety, stress and mood), alter TD (this was exploratory due to conflicting data), reduce salivary cortisol, be safe and tolerable, and be viewed as an acceptable form of treatment to people with AN.

## Materials and Methods

The protocol for this trial and supporting CONSORT checklist are available as supporting information (see S1 Protocol and S1 CONSORT Checklist). Ethical approval was given on 2^nd^ November 2012 by the London City & East National Research Ethics Service Committee (reference number: 12/LO/1525) and all participants gave written informed consent. The trial was registered prior to commencement (8^th^ February 2013 at www.controlled-trials.com, registration number: ISRCTN22851337). The raw data are available upon request to the authors.

### Participants

Using G*Power 3.1.9.2, a mixed 2 x 3 ANCOVA sample size calculation (i.e. incorporating a baseline covariate and accounting for interaction effects) was conducted based on our previous rTMS studies [34, 35]. This indicated that a total of 51 participants were required for an effect size of *d* = 0.90 with 80% power at two-sided *p* = 0.05. Accounting for a 5% dropout rate, a total of 27 people per group (i.e. total N = 54) were needed to obtain desired power.

Male and female participants >18 years of age were recruited from a specialist Eating Disorders Service and via the national eating disorder charity website www.b-eat.co.uk from 22^nd^ April 2013 until 28^th^ May 2014 (which was the planned length of time for study recruitment). Inclusion criteria were a current DSM-5 diagnosis of AN, established via referring clinicians and/or the Eating Disorder Diagnostic Scale (EDDS) [68] and a body mass index (BMI) between 14.5–18.5 kg/m^2^. Contra-indications to rTMS were checked with the TMS Adult Safety Screen (TASS) [69]. Further exclusion criteria were left-handedness, being on a dose of psychotropic medication that had not been stable for at least 14 days, pregnancy, and alcohol consumption (>3 units/day) and/or nicotine use (>15 cigarettes/day) [70].

### Procedures

**Fig 1:**
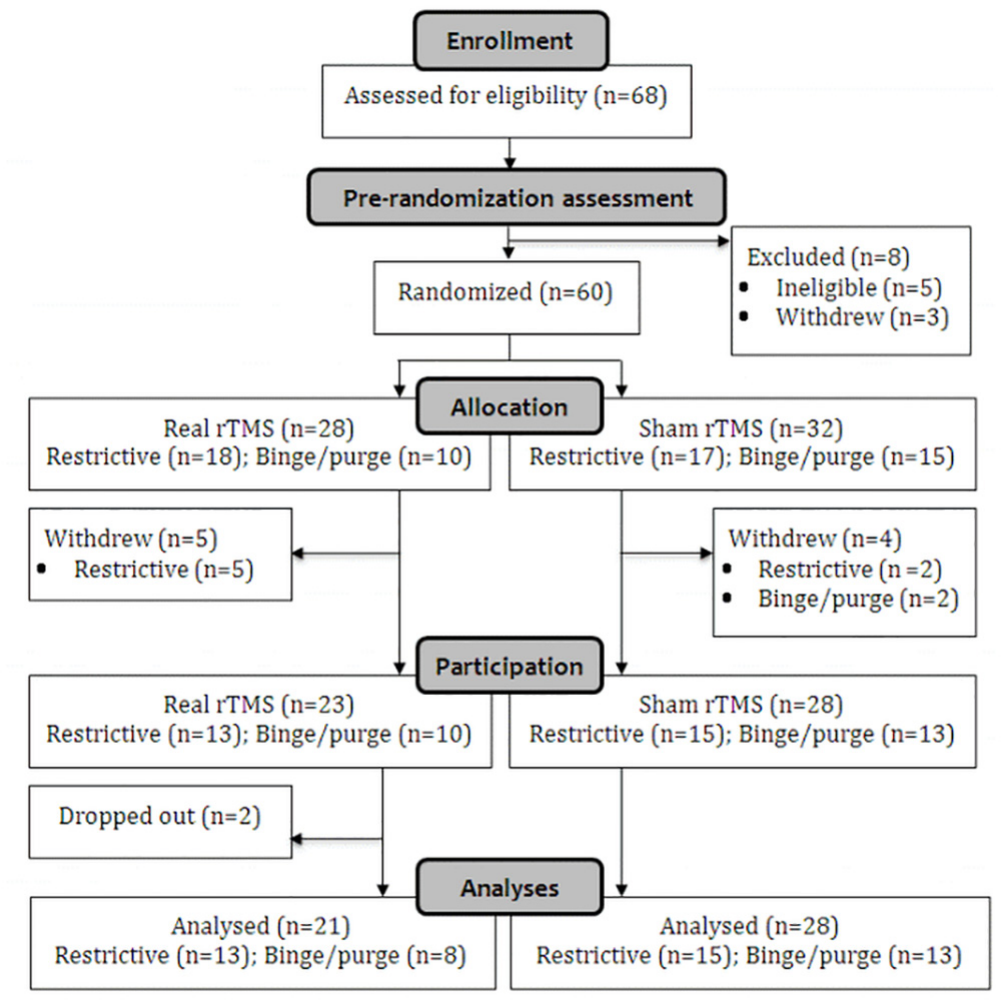
Recruitment and randomization CONSORT diagram.

This study followed recommendations for non-invasive brain stimulation trials [71]. As shown in the CONSORT diagram (Fig 1), participants’ eligibility was established using a pre-randomisation assessment which included; i) collection of demographic information (background, physical and psychiatric health, ED history, medications, alcohol/smoking behaviours), ii) administration of the TASS [69], iii) confirmation of diagnosis (via EDDS) and iv) assessment of psychiatric co-morbidity via the screening module of the Structured Clinical Interview for DSM-IV Axis I Disorders [72]. An independent researcher randomised eligible participants (*n* = 60) with STATA in a parallel design stratifying by AN-subtype using a random block design (block sizes of 2, 4, 6, and 8). The researcher administering rTMS was unblinded. To ensure allocation concealment, the independent researcher (randomiser) provided allocation details (stimulation type) via a participant ID-coded email to the rTMS administrator immediately before the rTMS session. Participants remained blind to stimulation type until the end of procedures, as did the researcher conducting assessments.

Nine participants withdrew post-randomization, e.g. due to feeling unwell. The remaining 51 completed procedures over one or two days and all study procedures took place at the Institute of Psychiatry, Psychology and Neuroscience, King’s College London. Participants underwent a structural magnetic resonance imaging scan [43]. This was used with Brainsight^®^ to neuronavigate the TMS coil to the l-DLPFC using Talaraich co-ordinates, x = -45, y = 45, z = 35 [73]. Participants were instructed to refrain from eating for at least one hour prior to the testing session.

**Fig 2:**
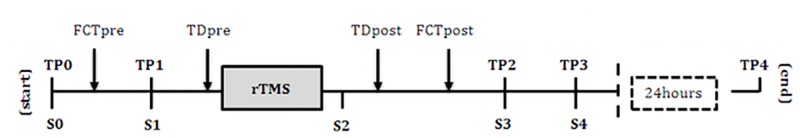
Testing protocol timeline. FCTpre & FCTpost: food challenge task before and after rTMS. TDpre & TDpost: temporal discounting task before and after rTMS. Collection of main visual analogue scales (VAS) at TP0 (pre-FCT); main VAS & additional VAS at TP1 (post-FCT/pre-rTMS) & TP2 (post-rTMS & FCT); main VAS only at TP3 (end of session) & TP4 (24 hours following). Saliva samples collected at S0 (pre-FCT), S1 (post-FCT/pre-rTMS), S2 (immediately after rTMS), S3 (post-rTMS/post-FCT) and S4 (end of session).

Fig 2 shows the testing protocol. All procedures were identical between groups, except for the rTMS. At the start (TP0), information on ED symptoms and general psychopathology was collected using the Eating Disorder Examination Questionnaire (EDE-Q version 6) [74] and the Depression Anxiety and Stress scales (DASS-21) [75]. Visual analogue scales (VAS; 10cm) assessing levels of urge to restrict, feeling full, feeling fat, stress and anxiety were administered (hereafter called ‘main’ VAS). A saliva sample (S0) was obtained using Salivettes^®^ along with measures of blood pressure (BP) and heart rate (pulse).

This was followed by the first administration of the food challenge task (FCTpre) [41]. The FCT required participants to watch a 2-minute film of people eating highly palatable foods (chocolate, nuts, crisps, biscuits) while the same foods were in the room, and then rate their perceived smell, taste, appearance and urge to eat these foods. This task serves as symptom provocation—it seeks to elicit AN-related experiences. At this point (TP1), the main VAS and ‘additional’ VAS regarding mood, calmness, hunger, general urge to eat, urge to binge, and urge to purge were completed. A saliva sample (S1) was collected and a computerised monetary TD task (TDpre) was administered [53].

The Brainsight^®^ neuronavigation and Magstim^®^ Rapid device (Magstim^®^, UK) were then used to establish participants’ motor threshold (MT) using peripheral electromyography; MT was defined as the minimum stimulation required to evoke 5 out of 10 motor evoked potentials greater than 50μV [76, 77]. Following MT measurement, BP and pulse were recorded. A real/sham coil was then used to administer neuronavigated rTMS; 5 second trains/55 second inter-train intervals, 10Hz, 110% MT, delivering 1000 pulses over 20 minutes to the l-DLPFC. Real and sham rTMS are identical to one another in terms of their set-up, duration and sound. Real rTMS emits a magnetic field that induces an electrical current in the brain (i.e. alters neural activity). Sham rTMS however, does not emit the magnetic field that can induce these changes in neural activity. Immediately after the 20^th^ train, BP and pulse were recorded, a saliva sample was collected (S2) and a 10cm VAS measuring discomfort experienced during the rTMS was completed.

The TD task was then re-administered (TDpost), followed by the FCT (FCTpost). Main and additional VAS were collected (TP2), followed by a saliva sample (S3). At the end of the session, the main VAS were repeated (TP3), a final saliva sample (S4) was collected and BP/pulse were recorded. Participants were phoned the next day for a 24-hour follow up (TP4) during which the main VAS were repeated, adverse events were discussed, and blinding was assessed and then revealed. Finally, participants were asked whether, if rTMS proved to be efficacious in AN, they would consider it as a treatment (i.e. 20 daily sessions).

#### Temporal discounting task

The TD task requires participants to choose between a smaller, variable amount of money (£0-£100) available immediately and a larger, fixed amount (£100) available after four different time delays (1 week, 1 month, 1 year and 2 years). The task is individually adjusted by an algorithm that changes the value of the immediate reward depending on the subject’s previous choices. This allows the calculation of each individual’s ‘indifference point’ i.e. the point at which the immediate reward is deemed equal to the fixed delayed amount [78, 79]. Using the indifference point values for each delay period, a hyperbolic decay function is calculated that describes the relationship between an individual’s subjective value of a reward as a function of its delay [53]: it involves a constant (*k*) that characterises an individual’s overall rate of discounting. Whilst the *k* value can be used as the main dependent variable, area under the curve (AUC) analysis provides a theoretically neutral account of TD. AUC is more appropriate for investigations with quantitative, inferential statistics, when *k* values are skewed and when relatively few time delays are used [80]. AUC values are between 0 and 1; smaller values reflect higher TD (choice impulsivity; higher sensitivity to delay) while larger values represent lower TD (less sensitivity to delay; temporal foresight).

### Analyses

The primary outcome variable was ‘core AN symptoms’ (computed by summation of VAS scores on urge to restrict, levels of feeling full and levels of feeling fat; maximum score of 30). All other outcomes were secondary. The effects of real versus sham rTMS on core AN symptoms and the five individual main VAS scores over time were evaluated using mixed ANCOVAs, i.e. both between and within group comparisons (group: real/sham rTMS x time: TP2, TP3 and TP4) controlling for scores at TP1 (TP1 scores were deemed our baseline as this was the time point immediately before the intervention). The effects of real versus sham rTMS on additional VAS (mood, calmness, hunger and urge to eat/binge/purge) were evaluated using mixed ANOVAs (group: real/sham rTMS x time: TP1, TP2).

In the analysis of the TD task, agreement between *k* and AUC were checked via correlation and then AUC was used as the outcome variable. The effect of real versus sham rTMS on AUC was evaluated using a mixed ANOVA (group: real/sham rTMS x time: TDpre, TDpost). Paired-sample t-tests were also used to look at the effects of real/sham rTMS within each AN subtype.

Salivary cortisol concentrations were analysed as previously described [67]. Correlation analyses between initial cortisol concentrations (S0) and psychopathology indices (EDEQ, DASS-21) were conducted. The effects of real versus sham rTMS on cortisol concentrations over time were evaluated using a mixed ANCOVA (group: real/sham rTMS x time: S2, S3 and S4) controlling for cortisol levels at S1. The effects of real versus sham rTMS on BP and pulse were evaluated using mixed ANCOVAs (group: real/sham rTMS x time: post-MT, post-rTMS, TP4) controlling for BP/pulse at TP0.

In this proof-of-concept study we were interested in the effects of rTMS in those who actually received their allocated intervention (real or sham rTMS) and completed all outcome assessments. Therefore, per-protocol statistical analyses were performed using IBM^®^ SPSS^®^ software (Version 22). When normality or other ANOVA assumptions were violated, non-parametric alternatives, log transformations or post-hoc bootstrapping methods with Bonferroni corrections were employed. All tests were two-tailed and the level of significance was set at *α* = 0.05. Partial eta squared (*η*^*2*^) and Cohen’s *d* effect sizes are reported for mixed ANOVAs/ANCOVAs and independent sample *t*-tests, respectively.

## Results

### Participant Flow and Baseline Characteristics

Two participants (both binge/purge AN) randomised to real rTMS withdrew following the first few trains of rTMS due to discomfort. Therefore, data from 49 females, randomised to real (*n* = 21) or sham rTMS (*n* = 28), were analysed. Table 1 shows demographic and clinical data. Of note, the median illness duration in the real rTMS group was 3 years shorter than the sham group but this was not statistically significant. There were no other significant between-group differences, except for the EDE-Q weight [*t*(47) = -2.05, *p* = 0.046, *d* = 0.60], and shape [*t*(47) = -2.06, *p* = 0.045, *d* = 0.60] subscale scores, which were higher in the sham group.

**Table 1:** Baseline characteristics (mean ± SD).

Participants (*n* = 49)	Real rTMS	Sham rTMS
	(*n* = 21)	(*n* = 28)
*Demographic information*		
AN subtype (R/BP)	13/8	15/13
Age	25.29 ± 6.88	27.68 ± 9.89
Illness duration; yrs	9.05 ± 7.02^	11.27 ± 8.01^
Meals/day	2.05 ± 0.92	2.29 ± 0.97
BMI	16.73 ± 1.59	16.38 ± 1.76
Smokers (yes/no)	6/21	9/28
On medication (yes/no)	12/21	22/28
*Eating disorder psychopathology*		
EDE-Q global	3.90 ± 1.26	4.43 ± 1.07
EDE-Q restraint	4.17 ± 1.68	4.41 ± 1.23
EDE-Q eating	3.63 ± 1.17	4.01 ± 1.32
EDE-Q weight*	3.48 ± 1.66	4.33 ± 1.28
EDE-Q shape*	4.32 ± 1.22	4.96 ± 0.98
*General psychopathology*		
DASS-21 total	29.67±13.65	33.93±12.32
DASS-21 depression	10.14 ± 5.98	12.00 ± 5.92
DASS-21 anxiety	7.05 ± 4.29	8.50 ± 4.37
DASS-21 stress	12.47 ± 4.99	13.43 ± 4.36

*sig *p* < .05. R: restrictive; BP: binge/purge;

^^^large variation/SD in illness duration, Real rTMS *Mdn* = 7 years, *range* = 3–35 years, Sham rTMS *Mdn* = 10 years, *range* = 1–38 years; EDE-Q scores: ≥ 2.8 indicate clinical severity; DASS-21 scores: 10+ depression, 6+ anxiety and 10+ stress indicate moderate/severe psychopathology.

### Salience of the Food Challenge Task

This was assessed via the main VAS before (TP0) and after (TP1) the first administration of the FCT. In the sample as a whole, the FCT significantly increased VAS anxiety scores [*t*(48) = -2.19, *p* = 0.034, *d* = 0.60] and there was a trend towards an increased urge to restrict [*t*(48) = -1.70, *p* = 0.095, *d* = 0.49]. By chance, at TP0 the sham group scored significantly higher on core AN symptoms [*t*(31.97) = -2.22, *p* = 0.034, *d* = 0.78] (real: *M* = 16.50, *SD* = 7.11; sham: *M* = 20.44, *SD* = 4.56) and feeling fat [*t*(36.21) = -2.62, *p* = 0.013, *d* = 0.87] (real: *M* = 5.37, *SD* = 2.96; sham: *M* = 7.40, *SD* = 2.26); however, these differences were not significant following the FCT, i.e. at TP1 (the rTMS intervention baseline).

### Primary outcome: core AN symptoms

**Fig 3:**
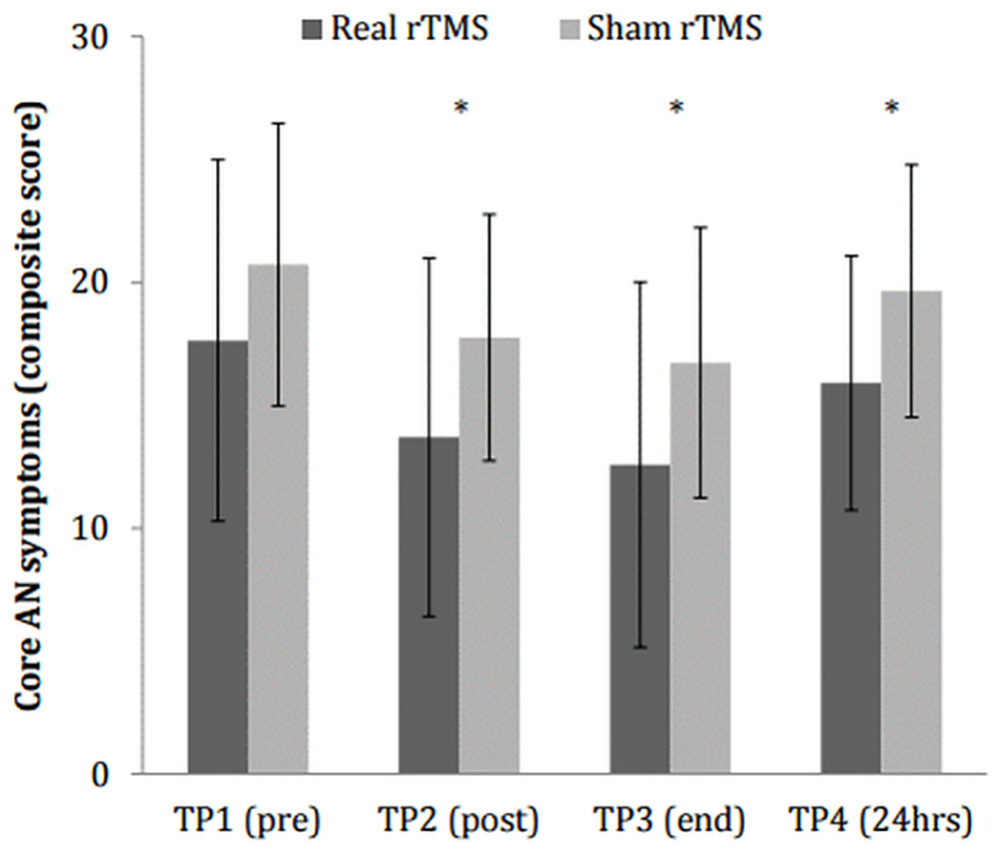
Mean ± SD of core AN symptoms pre-rTMS, post-rTMS, at the end of the session and after 24 hours. * *p* < .05 (prior to Bonferroni corrections). This composite core AN symptom outcome was composed of the urge to restrict (0–10), levels of feeling full (0–10) and levels of feeling fat (0–10) VAS and therefore scores can range from 0–30.

Mixed ANCOVA analyses controlling for pre-rTMS scores (TP1) showed no significant stimulation type x time interaction [*F*(1.35) = 0.14, *p* = 0.780, *η*^*2*^ = 0.00]; however, there was a main effect of time [*F*(1.35) = 13.58, *p* < 0.001, *η*^*2*^ = 0.23] and a trend towards group differences [*F*(1) = 3.86, *p* = 0.056, *η*^*2*^ = 0.08] (Fig 3). Those who received real rTMS reported lower levels of core AN symptoms after stimulation. Post hoc, bootstrapped comparisons suggested these group differences were significant at each of the three time points; TP2 (post-FCT2) [*t*(47) = -2.31, *p* = 0.030, *d* = 0.67], TP3 [*t*(47) = -2.24, *p* = 0.035, *d* = 0.65] and TP4 [*t*(47) = -2.51, *p* = 0.021, *d* = 0.73]. However, these differences were not significant following Bonferroni corrections for multiple comparisons.

### Secondary outcomes

Across the individual main VAS, mixed ANCOVA analyses controlling for pre-rTMS (TP1) scores demonstrated no interaction effects (Table 2). There was an effect of time on stress [*F*(1.55) = 8.40, *p =* 0.001, *η*^*2*^ = 0.15], anxiety [*F*(1.72) = 4.93, *p* = 0.013, *η*^*2*^ = 0.10], feeling full [*F*(1.28) = 21.89, *p* < 0.001, *η*^*2*^ = 0.32] and feeling fat [*F*(1.44) = 11.40, *p* < 0.001, *η*^*2*^ = 0.20]. There was also a trend for between group differences in levels of feeling fat [*F*(1) = 3.01, *p* = 0.089, *η*^*2*^ = 0.06]; reduced levels of feeling fat were reported following real rTMS. Post hoc, bootstrapped comparisons suggested that these group differences were significant at each of the three time points; TP2 [*t*(47) = -2.50, *p* = 0.030, *d* = 0.73], TP3 [*t*(47) = -2.82, *p* = 0.015, *d* = 0.82] and TP4 [*t*(47) = -2.27, *p* = 0.036, *d* = 0.66]. However, following Bonferroni corrections for multiple comparisons only differences at TP3 remained significant.

**Table 2:** Secondary outcomes; mean ± SD within each group across time points. AN-R: restrictive subtype; AN-BP: binge/purge subtype. Note: the values above are based on 10cm visual analogue scales (VAS). Scales were scored from ‘no urge to restrict/eat/binge eat/be sick or purge’ or ‘not feeling full/fat/hungry at all’ (0) to ‘extremely strong urge to restrict/eat/binge eat/be sick or purge’ or ‘feeling extremely full/fat/calm/hungry’ (10). The mood VAS was scored from ‘feeling extremely low’ (0) to ‘feeling extremely high’ (10) and the calmness VAS was scores from ‘feeling extremely calm’ (0) to ‘feeling extremely tense’ (10).

	Real rTMS (*n* = 21)				Sham rTMS (*n* = 28)			
	AN-R (*n* = 13), AN-BP (*n* = 8)				AN-R (*n* = 15), AN-BP (*n* = 13)			
‘Main’ VAS	TP1 (pre)	TP2 (post)	TP3 (end)	TP4 (24hrs)	TP1 (pre)	TP2 (post)	TP3 (end)	TP4 (24hrs)
Restrict	7.05 ±2.91	5.62 ± 3.00	5.18 ± 3.04	5.57 ± 2.48	7.38 ± 2.94	6.17 ± 2.39	5.76 ± 2.52	6.79 ± 2.11
Feeling full	5.03 ± 3.39	3.53 ± 2.80	3.33 ± 2.73	5.14 ± 2.90	6.08 ± 3.38	4.91 ± 3.18	4.44 ± 3.22	6.00 ± 2.70
Feeling fat	5.55 ± 3.23	4.55 ± 3.19	4.07 ± 3.41	5.19 ± 2.56	7.27 ± 2.61	6.68 ± 2.76	6.52 ± 2.68	6.86 ± 2.53
Anxiety	5.87 ± 3.32	4.25 ± 3.18	3.60 ± 3.27	4.38 ± 2.33	6.61 ± 3.02	5.00 ± 3.10	4.45 ± 2.94	5.46 ± 2.63
Stress	4.61 ± 3.21	3.87 ± 3.15	3.65 ± 3.26	4.24 ± 2.90	6.08 ± 2.70	4.52 ± 2.87	4.43 ± 2.90	5.21 ± 2.38
‘Additional’ VAS	TP1 (pre)		TP2 (post)		TP1 (pre)		TP2 (post)	
Mood	4.12 ± 1.88		4.88 ± 2.13		3.79 ± 1.58		4.56 ± 1.68	
Calmness	5.61 ± 3.16		4.01 ± 2.93		6.79 ± 2.14		5.21 ± 2.41	
Hunger	2.88 ± 2.68		4.27 ± 3.06		3.42 ± 3.54		5.02 ± 3.07	
Urge to eat	2.38 ± 2.60		3.93 ± 2.84		2.97 ± 3.37		4.43 ± 3.10	
Urge to binge eat	0.99 ± 1.95		1.12 ± 2.13		2.22 ± 3.24		1.91 ± 2.83	
Urge to be sick/purge	2.27 ± 3.14		1.10 ± 2.03		3.26 ± 3.68		2.60 ± 3.20	

### Additional VAS

There were no significant interactions across any of the additional VAS administered during the two FCT (Table 2). There was a significant effect of time [*F*(1) = 26.75, *p* < 0.001, *η*^*2*^ = 0.36] and a trend for group differences [*F*(1) = 2.91, *p* = 0.095, *η*^*2*^ = 0.06] on reported levels of calmness/tension. In addition, an effect of time on reported mood [*F*(1) = 13.32, *p* = 0.001, *η*^*2*^ = 0.22], hunger [*F*(1) = 15.29, *p* < 0.001, *η*^*2*^ = 0.25], urge to eat [*F*(1) = 13.11, *p* < 0.001, *η*^*2*^ = 0.22], and urge to be sick or purge [*F*(1) = 7.65, *p* = 0.008, *η*^*2*^ = 0.14] was found. There was no effect of time or group on participants’ urge to binge eat.

### Temporal discounting

**Fig 4:**
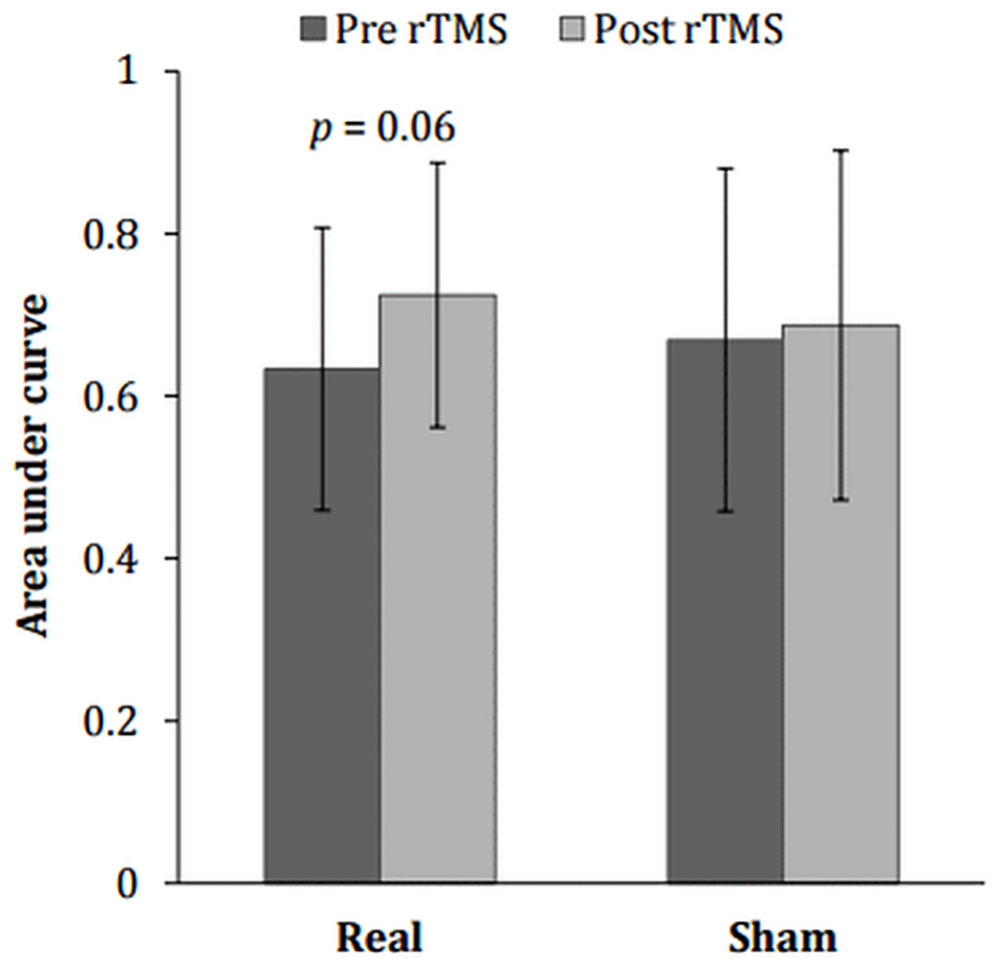
Mean + SD area under the curve (AUC) analysis of the temporal discounting (TD) task pre and post real/sham rTMS. Increased AUC represents reduced TD i.e. less steep TD/more prudent decision making.

Data from the TD task administered pre-rTMS were used for preliminary analyses. Data for the TD outcome variable, *k*, were highly skewed. Since Spearman’s correlation indicated agreement between *k* and AUC in measuring TD [*r*_*s*_ = -0.78, *p* < .001] AUC values were used in analyses. A mixed ANOVA indicated a trend towards a significant stimulation type x time interaction effect on AUC [*F*(1) = 3.71, *p* = 0.060, *n*^*2*^ = 0.07] (Fig 4). Paired sample *t*-tests suggested that real rTMS significantly increased AUC (i.e. reduced the rate of TD) [*t*(20) = -3.16, *p* = 0.005, *d* = 0.54], whilst sham rTMS had no effect on TD. Paired sample *t*-tests revealed that the effects of real rTMS on TD were significant within the restrictive AN subtype [*t*(12) = -2.91, *p* = 0.013, *d* = 0.54] but not within the binge/purge subtype.

### Cortisol

Salivary cortisol levels vary with time of day [81], therefore, all participants were tested in the afternoon. Forty-five complete data sets were available for analysis (19 real, 26 sham). As data were skewed, non-parametric correlation analyses were employed and log transformations were used in the mixed ANCOVA. Median baseline cortisol concentrations did not indicate hypercortisolaemia (6.63±5.09nmol/L). There were no significant associations between initial cortisol concentrations and psychopathology. There were also no initial (S0) differences in cortisol concentrations between real/sham groups, nor did the first FCT significantly alter cortisol levels. Controlling for pre-rTMS (S1) values, no significant interaction effects or effects of time or stimulation type were observed.

### Blinding

Although participants guessed stimulation type at a rate better than chance [*χ*^*2*^(1) = 4.59, *p* = 0.032], there were no significant differences between real/sham groups in the ability to correctly guess stimulation type; 43% who had real rTMS thought they had sham, while 29% who had sham rTMS thought they received real. Both groups had similar levels of certainty regarding how sure they were of which intervention they had received; real (*M* = 4.67, *SD* = 2.32), sham (*M* = 4.79, *SD* = 2.78). Researchers were not able to guess stimulation type at a rate better than chance. Levels of certainty in researchers were similar across groups; real (*M* = 2.24, *SD* = 2.32), sham (*M* = 2.43, *SD* = 2.30).

### Safety, tolerability and acceptability

Controlling for BP and pulse at TP0, there were no significant interaction, time or group effects. Real rTMS was experienced as more uncomfortable by participants than sham [*t*(46) = 6.33, *p* < 0.001, *d* = 1.87]; real (*M* = 5.51, *SD* = 2.48), sham (*M* = 1.38, *SD* = 2.04). However, there were no significant differences between real/sham groups in the number of physical complaints (typically feeling dizzy/dazed or having a headache) 24 hours after the session (5/21 real; 4/28 sham) or whether participants had to take painkillers (2/21 real, 1/28 sham). 90% of people who had real rTMS said that if rTMS proved to be efficacious in treating AN, they would consider having it as a treatment (i.e. 20 daily sessions).

## Discussion

In this proof-of-concept randomised controlled trial of rTMS in AN, individuals who received real rTMS (versus sham) tended to report reduced AN symptoms (statistical trend). However, given some improvements across this and other measures over time following both real and sham rTMS, there is also an indication of a placebo effect. The rate of TD was reduced following real (but not sham) rTMS (again, only at trend level), suggesting that rTMS may encourage more prudent decision making. Cortisol concentrations were not altered and rTMS was a safe, tolerable and acceptable procedure for people with AN.

Our findings, albeit only at trend level, are in line with existing evidence that neuromodulation may be able to alter AN symptomatology and intertemporal choice behaviour [40, 44, 60, 62, 82, 83]. Our uncontrolled pilot study reported that a single rTMS session temporarily reduced anxiety, levels of feeling full and feeling fat in participants with AN [41] and our case series also reported reductions within sessions across these symptoms [44]. Although existing data regarding TD in AN are not consistent, our modest findings regarding the effects of rTMS on TD are consistent with other research showing that the l-DLPFC is a key mediator of TD [59] and inhibitory/excitatory rTMS to the prefrontal cortex can increase/reduce TD, respectively [60, 62]. The lack of change in cortisol following rTMS replicates our previous findings in AN [41], but is not consistent with studies in healthy controls and other psychiatric groups, including BN [65–67].

We examined the effects of rTMS in AN across a range of psychological, neurocognitive and biological measures. We also used improved methodologies—the motor evoked potential method of estimating MT is more accurate and safer than other methods [84] and neuronavigated rTMS provides a more individualised, precise and effective method of targeting the DLPFC than older methods [73, 84, 85]. In addition, whilst participants were able to guess stimulation type better than chance, there was no difference between groups and thus blinding was partially successful.

This study is somewhat underpowered because of dropout. Our per-protocol analysis is also a limitation and may have led to statistical bias. The inclusion of a healthy comparison group would have been informative, particularly in terms of the TD findings. Furthermore, in relation to TD in AN, we did not measure or control for blood glucose levels. Whilst our choice of rTMS target, the l-DLPFC, is theoretically based, the optimal brain areas to target with neuromodulation in AN are unknown. The illness duration of the group who received real rTMS was, by chance but not significantly, shorter than those who received sham. On a neural level, illness duration may influence an individual’s responsivity to the effects of rTMS and thus it should be considered in future. By chance, initial ED and mood symptoms were higher in the sham group. Whilst this should be considered in the interpretation of results, there were no group differences immediately prior to real/sham rTMS and we are confident that our results are independent of this.

In terms of the mechanisms underlying therapeutic effects of rTMS in AN, our data suggest that real and sham rTMS do not differentially alter mood-related outcomes. Therefore, the trend for effects of rTMS on AN symptoms reported here may be independent of modulating emotion regulation abilities. Taken together, our findings of a tendency for real rTMS to reduce AN symptoms and improve intertemporal choice behaviour (i.e. cognitive control) suggest that transiently increasing PFC activity may temporarily improve AN symptoms relating to impaired inhibitory control [25, 26] and cognitive inflexibility [27]. More generally, this could imply that excitatory rTMS to the DLPFC in AN may (in the short-term) improve cognitive control mechanisms over symptoms that are described as rewarding, habitual, compulsive and ‘out-of-control’ (e.g. starvation, weight loss, exercise etc.) [21, 24, 86].

Given previous and current findings regarding rTMS in AN, together with the success of rTMS in treating other psychiatric disorders, further research is warranted. Proof-of-concept studies (such as this) are an important first step for non-invasive neuromodulation research in psychiatric conditions, although some have not demonstrated short-term psychological effects in other disorders [66]. Such single-session rTMS studies are limited in their validity and generalisability with regards to long-term therapeutic benefits and mechanisms of response. Preliminary evidence for the clinical utility of therapeutic rTMS in AN is encouraging [42–44]. Future research should include controlled therapeutic trials to establish/confirm the therapeutic efficacy of rTMS in AN. Neuroimaging modalities should also be used in order to probe disease mechanisms and identify biomarkers of response [87].

## Supporting Information

S1 Protocol Trial protocol. (DOCX)

S1 CONSORT Checklist CONSORT Checklist. (DOC)

S1 Dataset (SAV)
