# Supplementary material for: A Randomised Controlled Trial of Neuronavigated Repetitive Transcranial Magnetic Stimulation (rTMS) in Anorexia Nervosa
Source: PLoS One. 2016 Mar 23;11(3):e0148606. doi: 10.1371/journal.pone.0148606 (PMC4805273; doi:10.1371/journal.pone.0148606)
Supplement: S1 Protocol — (DOCX) [file pone.0148606.s001.docx]

**Repetitive Transcranial Magnetic Stimulation in Anorexia Nervosa**

**Aims and Objective**

The aim of this study is to examine whether repetitive Transcranial Magnetic Stimulation (rTMS) is effective in reducing the core symptoms of Anorexia Nervosa (AN). In a Randomised Control Trial (RCT) in adult outpatients with AN, one session of active, high frequency neuronavigated rTMS applied to the left dorsolateral prefrontal cortex (DLPFC) will be compared with sham rTMS in its ability to reduce AN patients’ desire to restrict food intake, food and body preoccupations, levels of stress and negative emotions, body interoceptive awareness and effect on executive functioning tasks. The objective of this comparison is to establish whether rTMS has therapeutic potential in the treatment of AN, a serious and debilitating psychiatric disorder.

**Background**

AN is a severe mental disorder with high levels of physical and psychological comorbidity and disability, and high mortality. In adults, response rates to treatment need to be improved as only 20 – 30% are in remission after one year of specialist treatment (NICE, 2004) [[1](#_ENREF_1)], and death, disability and poor quality of life are particularly common in the more chronic cases of AN [[2](#_ENREF_2)]. This underscores the need to obtain a better understanding of the aetiology of AN and to develop new treatments. Evidence is emerging that dysfunctional fronto-striatal circuits are involved in the aetiology of eating disorders (ED) [[3-5](#_ENREF_3)]. In AN, this is reflected by an interaction of poor behavioural self-control and preoccupation with food, eating, body shape and weight resulting in a desire to restrict intake. These behaviours are highly threatening and aversive.

Neuromodulatory techniques such as rTMS that directly alter brain functioning provide a promising avenue for exploring the neural basis of psychiatric conditions, including ED. rTMS is an increasingly accepted technique that is capable of either stimulating (high frequency) or inhibiting (low frequency) cortical areas. It has been used in a number of studies of psychiatric disorders and there is growing evidence for its beneficial effects [[6](#_ENREF_6), [7](#_ENREF_7)]. Application of high frequency rTMS to the skull in the region of the DLPFC has been shown to activate the areas underlying cerebral cortex [[8](#_ENREF_8)]. In addition, rTMS applied to the region of the left DLPFC is reported to result in increased dopamine neurotransmission in subcortical (striatal) brain structures [[9](#_ENREF_9), [10](#_ENREF_10)] and frontal cortical regions [[11](#_ENREF_11)], which has implications for reward circuits proposed to be altered in eating disorders [[4](#_ENREF_4), [12](#_ENREF_12)].

In two randomised sham-controlled trials, one rTMS session has been reported to reduce cue-elicited food cravings in healthy women who have high levels of food craving [[13](#_ENREF_13)] and also in a clinical sample of people with a bulimic eating disorder [[14](#_ENREF_14)]. Moreover, in the bulimic group, salivary cortisol levels were lower following real rTMS compared to sham treatment [[15](#_ENREF_15)], suggesting that the effects of rTMS on food craving may be related to changes in the HPA axis. Further promising results of rTMS in BN were found in a recent case report where rapid and unexpected remission of BN occurred in a patient being treated with rTMS primarily for comorbid depression [[16](#_ENREF_16)].

Support for the use of rTMS in AN comes from neuroimaging studies and a case report where rTMS was successfully used to treat comorbid depression in an individual with AN [[17](#_ENREF_17)]. Since the studies in food craving and bulimia [[13](#_ENREF_13), [14](#_ENREF_14)], an uncontrolled pilot study investigating whether one session of high frequency rTMS delivered to the left DLPFC showed a reduction in eating disorder symptoms in ten AN patients following exposure to visual and real food stimuli [[18](#_ENREF_18)]. Safety and tolerability of rTMS were assessed and showed that rTMS is a safe and well-tolerated technique. Subjective experiences related to the eating disorder (e.g. urge to restrict, feeling full etc.) were assessed before and after rTMS. Results suggest that it reduced levels of feeling full, feeling fat and feeling anxious. Thus, rTMS may reduce core symptoms of AN.

A key limitation of previous rTMS studies is the relative inaccuracy of the procedure used to locate the anatomical target of the rTMS (the left DLPFC in the case of AN). Previous studies employed the standard “5cm anterior method” of locating the left DLPFC [[14](#_ENREF_14), [18-20](#_ENREF_18)]: however, a growing literature suggests that neuronavigation techniques are more accurate and thus may elicit greater therapeutic effects [[21-24](#_ENREF_21)]. Therefore, we propose to conduct the first RCT of rTMS in AN, using neuronavigation to obtain a high degree of accuracy for the localisation of the stimulus.

**Hypotheses**

Based on our previous pilot study [[18](#_ENREF_18)] and the ability of rTMS to stimulate underlying cortical areas, we hypothesise that; in people with AN who are presented with highly appetizing food stimuli real, high-frequency neuronavigated rTMS applied to the left DLPFC, compared to sham rTMS, will lead to a reduction in a) wanting to restrict food intake; b) perceived stress and negative emotions; c) food, eating and body related preoccupations; and d) salivary cortisol levels. The intervention will also lead to an improvement in a) body/interoceptive awareness; and b) an executive functioning task. Lastly, it will lead to an increase in food intake.

**Methodology**

*Experimental Design.* Sixty-four participants will be randomized to receive either real or sham rTMS resulting in 32 individuals in each treatment arm, in a parallel group double-blind design. The randomization will be stratified, based on the AN-subtype: restricting or binge/purging type.

*Statistics.* A sample size calculation based on the effect size found in our previous rTMS in bulimic disorders study in which we applied a similar rTMS paradigm [[14](#_ENREF_14)], indicates that this will allow detection of significant differences (p<0.05) between groups with 80% power. This takes into account an estimated 5% drop-out. As the experimental design only requires a one day attendance session, we do not expect there to be a large drop-out rate.

The primary outcome measures are the scores on the VAS scales a) ‘stress’, b) ‘urge to restrict’, c) ‘anxiety’, d) ‘urge to exercise’, e) ‘feeling full’ and f) ‘feeling fat’ after real or sham rTMS. The secondary outcome measures are a) all other VAS scales, b) salivary cortisol levels, c) consumption of smoothie, d) eating behaviours in the 24 hours post rTMS/sham, e) effects on the cognitive task, f) percentage of people dropping out, g) the percentage of people reporting adverse effects and h) blinding success. Both ‘completer’ and ‘intention-to-treat’ analyses will be conducted. Group difference on primary outcomes (post rTMS) will be tested with an analysis of covariance, including the pre-rTMS measure as a covariate.

*Participants.* We will recruit 64 adults with a DSM-IV diagnosis of AN or Eating Disorder Not Otherwise Specified-AN type (EDNOS-AN). Men and women will be included in the study providing they have one of these diagnoses and are 18 years or older. Exclusion criteria are; being on a dose of psychotropic medicine that has not been stable for at least 14 days, pregnancy and excessive nicotine use or substance dependence. Patients will be recruited from the Eating Disorders Unit in the South London and Maudsley NHS Foundation Trust (SLAM). The SLaM service sees approximately 250 new adult (over the age of 18) AN patients per annum. Further participants will also be recruited from circular email and poster advertisements in the University and via the Beat website (Eating Disorders Association).

*Methods* (see Table 1)*.* After screening for inclusion and exclusion criteria, eligible participants will attend for a single visit during which all study procedures will take place. The structure of the visit/study protocol is set out in Table 1. Providing written consent has been obtained and all other safety measures have been assessed, participants will be required to complete the TMS Adult Safety Screen Questionnaire. Demographic information (including height and weight) will be obtained in addition to completion of the Eating Disorder Examination Questionnaire (EDE-Q), Structural Clinical Interview for DSM Disorders (SCID) and the short, 21 item Depression, Anxiety and Stress Scale (DASS-21). After confirming that the participant is safe to undergo a Magnetic Resonance Imaging scan (MRI), they will then undergo a structural MRI in order to obtain an individualised brain scan. This will be used to locate the target of the rTMS, the left DLPFC using neuronavigation software called Brainsight™.

Following the MRI, participants will be given a break of approximately two to three hours. This is in order to allow for the scan to be processed, downloaded and mapped onto the Brainsight™ software and for the participant to have a lunch break. Participants will be instructed not to eat anything one hour prior to the time agreed to meet for the second part of testing. This is in order to ensure that individuals are appropriately satiated by the time they are presented with the food tasks (description to follow).

After the break, the first of four saliva samples will be taken, along with blood pressure and pulse measurements. Baseline assessments to evaluate mood, anxiety and stress (via DASS-21), Body Awareness (BAS) and feeling full, feeling fat, urge to restrict and urge to exercise (via visual analogue scales; VAS) will then be obtained. A neuropsychological task that engages the frontal cortex will be administered.

Participants will then undertake the first of two ‘Food Challenge Tasks’ (FCT). This is a behavioural measure that we developed previously [[13](#_ENREF_13), [14](#_ENREF_14)]. During this task, a short film is shown of people eating highly appetitive foods. This is followed by exposure to real foods that are highly palatable (e.g. chocolate). Participants are then requested to rate, on the Food Challenge Task State VAS (FCT-S), emotional states such as stress and anxiety, level of preoccupation with food, body weight and shape, and attitudes toward food intake (e.g. urge to restrict intake). Salivary cortisol samples and the baseline VAS assessments will also be collected again to assess saliency of the FCT.

Next, through mapping of the abductor pollicis brevis site, the intensity of the rTMS will be acquired by obtaining the individuals motor threshold (MT) which represents membrane-related excitability of cortical axons. Using the Motor Evoked Potential Method (MEPM), the MT is established by determining the minimum stimulator output intensity required to obtain 5 out of 10 motor evoked potentials (MEP) greater than 50µV [[25](#_ENREF_25)]. Based on this measurement, one session of high frequency rTMS (10Hz) at 110% of the individual’s MT, consisting of twenty 5-seconds trains with 55-seconds inter-train intervals will be delivered to the left DLPFC. Unlike previous studies whereby the left DLPFC was localized using the standard 5cm anterior method [[14](#_ENREF_14)], we propose to use the individual structural MRI scans acquired earlier in the day in order to locate the left DLPFC. To ensure safety and tolerance, blood pressure and pulse will be taken every five trains and level of discomfort will be assessed after train one, two, three, five, ten, fifteen and twenty.

A Magstim Rapid device (Magstim®, UK) will be used to administer real and sham rTMS. All of the aforementioned protocol, including establishing each individuals MT will be identical between groups. The sham stimulation will be given at the same location and frequency as the real rTMS however a sham coil will be used. All of these procedures and parameters are in accordance with the current safety and application guidelines for rTMS [[26](#_ENREF_26)]. The rTMS equipment is available in the Institute of Psychiatry and we will be collaborating with Professor Anthony David who is in charge of this equipment.

Immediately after the rTMS/sham session, the neuropsychological task will be repeated. Following this, the second (but identical) FCT will be administered and the associated assessment of levels of emotions, preoccupations, and attitudes toward food will be measures on both the FCT-S and VAS. A third saliva sample will then be taken, the BAS and DASS-21 will be completed again by the participant.

At the end of these tasks an assessment of discomfort, acceptability and any adverse side effects will be completed. A fourth saliva sample and final VAS will then be completed shortly followed by the participant being asked to consume as much as possible of a smoothie drink from a choice of three available flavours. A final measure of blood pressure and pulse will then be taken, and participants will be asked if it is okay for one of us to contact them tomorrow to see how they are doing and ask a few questions relating to the 24 hours following the testing session. They will be reimbursed for their time, efforts and travel (£50).

One of the researchers will call the participant at an agreed time the following day. The VAS will be repeated in relation to the past 24 hours and any further side effects will be investigated. Participants will then be asked to guess which stimulation (real/sham) they think they received, and how sure they are of this decision. After guessing stimulation type, participants will be informed of the actual stimulation they received. Finally, before being thanked for their time and involvement in the study, participants will be asked that, if it were available would they consider participating in longer, therapeutic trial of rTMS.

**Table 1: RCT Protocol**

| **Timeline** | **Duration** | **TP** | **Tasks/Assessments** | **Technician** |
| --- | --- | --- | --- | --- |
| ± 7 hours | 20 mins | Screening | Demographic information, inclusion/ exclusion criteria, TMS safety questionnaire, EDDS, SCID | Un-blinded |
|  | 10 mins | Baseline | DASS-21, EDE-Q |  |
|  | 1hr | MRI | MRI safety questionnaire, MRI scan |  |
|  | 2-3 hours | **BREAK**  (no eating 1 hour prior to meeting for next stage of testing) | | |
|  | 1 hour | **TP0** | Saliva (I) | Un-blinded |
|  |  |  | Blood pressure & pulse (I) |  |
|  |  |  | VAS (I), BAS (I) | Blinded |
|  |  |  | Temporal Discounting Task (I) |  |
|  |  | ***Food Challenge Task (FCT 1)*** | |  |
|  |  | **TP1** | Food tasting/rating (VAS II & FCT-S I) |  |
|  |  |  | Saliva (II) | Un-blinded |
|  | 45 mins | **~ REAL/SHAM rTMS SESSION ~**  Motor Threshold (MT) established  Real/sham rTMS applied, Saliva (III)  Blood pressure and pulse  Level of discomfort | |  |
|  | 1 hour | **TP2** | Temporal Discounting Task (II) | Blinded |
|  |  | ***Food Challenge Task (FCT 2)*** | |  |
|  |  | **TP2** | Food tasting/rating (VAS III & FCT-S II) |  |
|  |  |  | Saliva (IV) |  |
|  |  |  | BAS (II) |  |
|  |  | **TP3** | Tolerance, acceptability and side effects |  |
|  |  |  | Saliva (V), VAS (IV) |  |
|  |  |  | Smoothie |  |
|  |  |  | Blood pressure & pulse (VI) |  |
|  | 15 mins | **TP4**  24hr call | VAS (V) | Un-blinded |
|  |  |  | Test of blinding |  |

**References**

1. Schmidt, U., et al., *A Randomised Controlled Trial of a Novel Trait Focused Treatment in Adults with Anorexia Nervosa.* British Journal of Psychiatry., 2012.

2. Steinhausen, H.C., *The outcome of anorexia nervosa in the 20th century.* American Journal of Psychiatry, 2002. **159**(8): p. 1284-1293.

3. Brooks, S.J., et al., *Differential neural responses to food images in women with bulimia versus anorexia nervosa.* PloS one, 2011. **6**(7): p. e22259.

4. Kaye, W., J. Fudge, and M. Paulus, *New insights into symptoms and neurocircuit function of anorexia nervosa.* Nature Reviews Neuroscience, 2009. **10**(8): p. 573-584.

5. Marsh, R., T.V. Maia, and B.S. Peterson, *Functional disturbances within frontostriatal circuits across multiple childhood psychopathologies.* American Journal Psychiatry, 2009. **166**(6): p. 664-74.

6. Slotema, C.W., et al., *Should we expand the toolbox of psychiatric treatment methods to include Repetitive Transcranial Magnetic Stimulation (rTMS)? A meta-analysis of the efficacy of rTMS in psychiatric disorders.* The Journal of Clinical Psychiatry, 2010. **71**(7): p. 873-884.

7. Wassermann, E.M. and S.H. Lisanby, *Therapeutic application of repetitive transcranial magnetic stimulation: a review.* Clinical Neurophysiology, 2001. **112**(8): p. 1367-1377.

8. Nahas, Z., et al., *Brain Effects of TMS Delivered Over Prefrontal Cortex in Depressed AdultsRole of Stimulation Frequency and Coil–Cortex Distance.* The Journal of Neuropsychiatry and Clinical Neurosciences, 2001. **13**(4): p. 459-470.

9. Pogarell, O., et al., *Acute prefrontal rTMS increases striatal dopamine to a similar degree as D-amphetamine.* Psychiatry Research: Neuroimaging, 2007. **156**(3): p. 251-255.

10. Pogarell, O., et al., *Striatal dopamine release after prefrontal repetitive transcranial magnetic stimulation in major depression: preliminary results of a dynamic [123I] IBZM SPECT study.* Journal of Psychiatric Research, 2006. **40**(4): p. 307-314.

11. Cho, S.S. and A.P. Strafella, *rTMS of the left dorsolateral prefrontal cortex modulates dopamine release in the ipsilateral anterior cingulate cortex and orbitofrontal cortex.* PloS one, 2009. **4**(8): p. e6725.

12. Kaye, W., et al., *Neurocircuity of eating disorders.* Current Topics in Behavioural Neurosciences, 2011. **6**: p. 37-57.

13. Uher, R., et al., *Effect of left prefrontal repetitive transcranial magnetic stimulation on food craving.* Biological Psychiatry, 2005. **58**(10): p. 840-842.

14. Van den Eynde, F., et al., *Repetitive transcranial magnetic stimulation reduces cue-induced food craving in bulimic disorders.* Biological Psychiatry, 2010. **67**(8): p. 793-795.

15. Claudino, A., et al., *Repetitive transcranial magnetic stimulation reduces cortisol concentrations in bulimic disorders.* Psychological Medicine, 2011. **41**(1): p. 1329-1336.

16. Downar, J., et al., *Unanticipated Rapid Remission of Refractory Bulimia Nervosa, during High-Dose Repetitive Transcranial Magnetic Stimulation of the Dorsomedial Prefrontal Cortex: A Case Report.* Frontiers in Psychiatry, 2012. **3**: p. 30.

17. Kamolz, S., et al., *Transkranielle Magnetstimulation gegen komorbide Depression bei Anorexie.* Der Nervenarzt, 2008. **79**(9): p. 1071-1073.

18. Van den Eynde, F., et al., *Repetitive transcranial magnetic stimulation in anorexia nervosa: A pilot study.* European Psychiatry, 2011.

19. Van den Eynde, F., et al., *Handedness, repetitive transcranial magnetic stimulation and bulimic disorders.* European Psychiatry, 2010.

20. Broadbent, H.J., et al., *Blinding success of rTMS applied to the dorsolateral prefrontal cortex in randomised sham-controlled trials: A systematic review.* World Journal of Biological Psychiatry, 2011. **12**(0): p. 1-9.

21. Sparing, R., M.D. Hesse, and G.R. Fink, *Neuronavigation for transcranial magnetic stimulation (TMS): where we are and where we are going.* Cortex, 2010. **46**(1): p. 118-120.

22. Ahdab, R., et al., *Comparison of "standard" and "navigated" procedures of TMS coil positioning over motor, premotor and prefrontal targets in patients with chronic pain and depression.* Neurophysiologie Clinique/Clinical Neurophysiology, 2010. **40**(1): p. 27-36.

23. Herwig, U., et al., *Transcranial magnetic stimulation in therapy studies: examination of the reliability of “standard” coil positioning by neuronavigation.* Biological Psychiatry, 2001. **50**(1): p. 58-61.

24. Lefaucheur, J.P., *Why image-guided navigation becomes essential in the practice of transcranial magnetic stimulation.* Neurophysiologie Clinique, 2010. **40**(1): p. 1-5.

25. Rothwell, J.C., et al., *Magnetic stimulation: motor evoked potentials. The International Federation of Clinical Neurophysiology.* Electroencephalography and Clinical Neurophysiology. Supplement, 1999. **52**: p. 97-103.

26. Rossi, S., et al., *Safety, ethical considerations, and application guidelines for the use of transcranial magnetic stimulation in clinical practice and research.* Clinical Neurophysiology, 2009. **120**(12): p. 2008-39.
